# Wilms' tumour 1 can suppress *hTERT* gene expression and telomerase activity in clear cell renal cell carcinoma via multiple pathways

**DOI:** 10.1038/sj.bjc.6605878

**Published:** 2010-09-14

**Authors:** R T Sitaram, S Degerman, B Ljungberg, E Andersson, Y Oji, H Sugiyama, G Roos, A Li

**Affiliations:** 1 Department of Medical Biosciences and Pathology, Umeå University, Umeå, Sweden; 2 Department of Perioperative Sciences, Urology and Andrology, Umeå University, Umeå, Sweden; 3 Department of Cancer Stem Cell Biology, Osaka University Graduate School of Medicine, Osaka, Japan; 4 Department of Functional Diagnostic Science, Osaka University Graduate School of Medicine, Osaka, Japan; 5 Department of Medical Biosciences and Clinical Chemistry, Umeå University, Byg 6M, 2nd floor, Umeå SE-90185, Sweden

**Keywords:** renal cell carcinoma, *WT1*, *hTERT*, telomerase activity and pathways

## Abstract

**Background:**

Wilms' tumour 1 (*WT1*) gene was discovered as a tumour suppressor gene. Later findings have suggested that WT1 also can be oncogenic. This complexity is partly explained by the fact that WT1 has a number of target genes.

**Method:**

WT1 and its target gene human telomerase reverse transcriptase (*hTERT*) were analysed in clear cell renal cell carcinoma (ccRCC). *In vitro* experiments were performed to examine the functional link between *WT1* and *hTERT* by overexpression of WT1 isoforms in the ccRCC cell line, TK-10.

**Results:**

*WT1* demonstrated lower RNA expression in ccRCC compared with renal cortical tissue, whereas *hTERT* was increased, showing a negative correlation between *WT1* and *hTERT* (*P*=0.005). These findings were experimentally confirmed *in vitro*. The WT1 generated effect on *hTERT* promoter activity seemed complex, as several negative regulators of *hTERT* transcription, such as *SMAD3*, *JUN* (*AP-1*) and *ETS1*, were activated by WT1 overexpression. Downregulation of potential positive *hTERT* regulators, such as *cMyc*, *AP-2α*, *AP-2γ*, *IRF1*, *NFX1* and *GM-CSF*, were also observed. Chromatin immunoprecipitation analysis verified WT1 binding to the *hTERT*, *cMyc* and *SMAD3* promoters.

**Conclusion:**

The collected data strongly indicate multiple pathways for *hTERT* regulation by WT1 in ccRCC.

Renal cell carcinoma (RCC) accounts for 3% of adult malignancies and has a high mortality rate. The three main subtypes of RCC are clear cell (ccRCC), papillary and chromophobe RCC (Kovacs *et al*, 1997). Clear cell RCC, representing 75–80% of all RCCs, is characterised by loss of 3p14.2-p25, duplication of 5q22 and deletions of chromosomes 6q, 8p, 9p and 14q (Kovacs, 1996).

The Wilms' tumour 1 (*WT1*) gene is an important regulator of cell growth and development with expression in the developing embryo kidney, adult urogenital system, central nervous system and in the hematopoietic system (reviewed in Scharnhorst *et al* (2001)). In 1990, *WT1* was discovered as a tumour suppressor gene in Wilm's tumour, a childhood kidney neoplasm (Committe TNWTS, 1991; Haber *et al*, 1990). This gene encodes a 49–52 kDa protein with an N-terminal domain involved in RNA/protein interactions critical for its transcriptional regulatory function (Call *et al*, 1990). The C-terminal domain is involved in RNA and protein interactions and harbours four zinc-fingers and two nuclear localisation signals, which permit binding to target DNA sequences (Bruening *et al*, 1996). By two main alternate splicing events including splicing of exon 5 (17 amino acids) and exon 9 (three amino acids: lysine, threonine and serine (KTS)), four WT1 protein isoforms are produced designated by the presence or absence of exon 5/KTS as A (−/−), B (+/−), C (−/+) and D (+/+) (Haber *et al*, 1991). WT1A (−/−) was demonstrated to induce morphological changes and promote cell migration and invasion in ovarian cancer cells (TYK) (Jomgeow *et al*, 2006). In osteosarcoma cell lines, induction in programmed cell death was preferentially mediated by WT1B (+/−) through transcriptional repression of the *EGFR* gene (Haber *et al*, 1996). WT1D (+/+) can cause a morphological transition from an epithelial to a more mesenchymal phenotype (Burwell *et al*, 2007). As suggested by the presence of zinc-fingers, WT1 is a potent transcriptional regulator. A large number of genes coding for growth factors (e.g., TGF-*β*, CSF-1), growth factor receptors (e.g., insulin R, IGF-IR, EGFR), transcription factors (e.g., EGR, WT1, cMyc, Pax2, Dax-1 and Sry) and other proteins (e.g., ODC, MDR1, Hsp70, p21, Bcl-2) have been identified as WT1 target genes (reviewed in Ariyaratana and Loeb (2007); Yang *et al* (2007)). Also, the WT1 protein has interacting partners such as p53 and STAT3, which can modulate WT1 transcription functions (Maheswaran *et al*, 1993; Rong *et al*, 2006).

Telomerase activation is a key event during immortalisation and malignant transformation contributing to telomere stabilisation and extended replicative capacity. Human telomerase reverse transcriptase (hTERT) is a key component of the telomerase complex regulated on several levels. The *hTERT* promoter contains methylation-accessible CpG islands and its methylation status has been associated with *hTERT* transcriptional repression (Devereux *et al*, 1999; Chatagnon *et al*, 2009). The *hTERT* core promoter and sequences upstream contain numerous binding sites for positive and negative regulators of transcription suggesting a complex regulation (Takakura *et al*, 1999). A number of factors can directly or indirectly regulate the *hTERT* promoter, including activators (cMyc, Sp1, ER, HIF-1*α*, E6 protein, activating enhancer-binding protein (AP-2) and so on), as well as repressors (WT1, AP-1, p53, p73, MZF, pRb, E2F and so on) (Cukusic *et al*, 2008). We have recently shown that the PI3K pathway is one significant road for *hTERT* regulation in ccRCC and that cMyc binding to the *hTERT* promoter seemed important for this control (Sitaram *et al*, 2009).

Oh *et al* (1999) identified WT1 as a transcriptional repressor of *hTERT* in virally transformed human embryonic kidney 293 cells, but the WT1 regulation seemed to be cell type specific. In this study, we could demonstrate negative associations between *WT1* and *hTERT* and between *WT1* and *cMyc* in clinical ccRCC samples, data that were verified by cell line transfection experiments. Forced expression of WT1 in the ccRCC TK-10 cell line reduced *hTERT* mRNA levels and telomerase activity by direct WT1 binding to the *hTERT* promoter, but also by affecting several genes known to regulate *hTERT* transcription. Our results suggest that *WT1* can act as a tumour suppressor in ccRCC via multiple pathways leading to downregulation of *hTERT.*

## Materials and methods

### Tissue samples

We performed the study on a total of 73 ccRCC tumour specimens and 26 tumour-free renal cortical tissue samples. The tissue specimens were collected between February 1988 and December 2003 under a protocol approved by the Human Ethics Committee of the Medical Faculty, Umeå University. Each patient participated after providing informed consent and during the later years also with informed and signed consent. All pathology specimens were reviewed by pathologists according to (Skinner *et al*, 1971). Tumour stages were classified according to the TNM classification 2002 (Greene and Sobin, 2002). Follow-up medical records of the patients were retrospectively updated by surgical urologists and were used for survival analysis.

### RNA extraction and reverse transcription

Total RNA was isolated from snap-frozen tumour specimens and tumour-free renal cortical tissue using the TRIzol method (Invitrogen, Stockholm, Sweden). cDNA was prepared by reverse transcription with the Superscript II Reverse transcriptase kit according to the manufacturers' protocol (Invitrogen).

### Genome-wide gene expression array

Total RNA (200 ng) of each sample was used to produce cRNA according to the provided protocol of the Illumina Total Prep RNA Amplification Kit (Ambion Inc., Austin, TX, USA).

A total of 750 ng biotinylated cRNA was used for hybridisation to a human HT12 Illumina Beadchip gene expression array according to the manufacturers' protocol (Illumina, San Diego, CA, USA). The arrays were scanned using the Illumina Bead Array Reader. For data analysis and normalisation the Illumina BeadStudio 3.2 software was used. Cell signalling pathway and network analysis was carried out with the Metacore software (GeneGo Inc, St Joseph, MI, USA). Samples were normalised by the quantile algorithm, genes with signal below background levels were excluded, and differentially expressed genes were identified by fold change calculations and with the Illumina custom differential expression algorithm (described in the Illumina Gene Expression Module user guide) to identify ⩾2-fold and statistically (*P*<0.01) differently expressed genes.

### Real-time PCR

Quantitative real-time PCR (qRT–PCR) using TaqMan technology was performed for *WT1* expression. Following primers and probe given, a 119-bp product was used to detect *WT1* mRNA levels. Forward primer: 5′-GCTATTCGCAATCAGGGTTACAG-3′ (located on exon 1/2), reverse primer: 5′-TGGGATCCTCATGCTTGAATG-3′ (located on exon 2); and TaqMan probe: 5′-CACACGCCCTCGCACCATGC-3′ (located on exon 2). The *β-actin* gene was used for normalisation of cDNA templates, and sequences of the primes and probe were previously described (Inoue *et al*, 1997). The qRT–PCR reaction was initiated with 2 min of incubation at 50°C and then for 10 min at 95°C, followed by 45 cycles of denaturation at 95°C for 15 s and annealing at 60°C for 2 min. Standard curves were generated by 10-fold dilutions of plasmid DNA containing the insert *WT1* or *β-actin* genes.

The expression of *hTERT* mRNA was measured using the Light Cycler TeloTAGGG *hTERT* quantification kit (Roche Diagnostics, GmbH, Mannheim, Germany). By using a reference standard curve provided from the qRT–PCR kit, the relative *hTERT* mRNA expression (with reference to housekeeping gene, porphobilinogen deaminase (*PBGD*)) was calculated as previously described (Sitaram *et al*, 2009). For *c-Myc*, mRNA quantification was performed by qRT–PCR using SYBR green I technology (Roche Diagnostics) as previously described (Sitaram *et al*, 2009). Values of target gene expression were calculated with template normalised to house keeping gene *PBGD*.

*SMAD3*, *ETS1* and *AP-2α* were analysed by TaqMan assays according to manufacturer's protocol with the TaqMan universal PCR mastermix and run on the ABI Prism 7000 Sequence Detection System, *SMAD3* (Hs00232222_m1), *ETS1* (Hs_00901425_m1) and *AP-2α* (Hs_01029410_m1) (Applied Biosystems, Foster City, CA, USA). cDNA from the T-cell lymphoma cell line (CCRF) was used to generate the standard curves. Collected data were normalised to *β-actin* as described above.

### Cell culture, plasmid and transient WT1 A (−/−) and D (+/+) transfection

TK-10 cell line with undetectable endogenous WT1 protein was derived from a primary ccRCC tumour (provided by Dr Xu, Karolinska Institutet, Stockholm, Sweden) and was used for transfection experiments. The cells were maintained in 1 × DMEM (Gibco, Stockholm, Sweden) containing 10% fetal calf serum in 5% CO2 at 37°C. pcDNA 3.1(+) vectors (Invitrogen, Carlsbad, CA, USA) containing *WT1* variant A (−/−) or D (+/+) were constructed as described previously (Jomgeow *et al*, 2006).

TK-10 cells were transiently transfected with 1 *μ*g per well (1 × 10^5^ cells) of *WT1A* or *WT1D* pcDNA 3.1(+) vectors using FuGENE 6 (Roche Diagnostic Corp, Indianapolis, IN, USA). pcDNA 3.1(+) vector without insert of *WT1*A or *WT1*D was used as control. All cells were collected 24 and 48 h after transfection for further analysis.

### ChIP analysis

Chromatin immunoprecipitations (ChIPs) were performed using the Chromatin Immunoprecipitation Kit (Upstate Millipore, Billerica, MA, USA). Approximately 2–3 × 10^6^ WT1-transfected TK-10 cells were crosslinked with 1% formaldehyde, followed by glycine to quench unreacted formaldehyde. Chromatin was sonicated on ice to shear crosslinked DNA to about 200–1000 bp in length using a Sonifier ultrasonic cell disruptor (Branson, Danbury, CT, USA) with 12 × 10 s pulses. The sheared chromatin was resuspended in dilution buffer and 1% of the chromatin was removed as input, followed by immunoprecipitation using protein G magnetic beads with 2 *μ*g of either anti-WT1 (C-19) antibody (Santa Cruz Biotechnology Inc, Santa Cruz, CA, USA,) or normal rabbit IgG (Cell Signalling Technology Inc, Danvers, MA, USA) at 4°C overnight with rotation. After the reversal of crosslinks by incubation in ChIP elution buffer containing proteinase K at 62°C for 2 h, DNA was purified using spin columns.

The PCRs containing 2 *μ*l of the immunoprecipitated DNA or input chromatin, primers and AmpliTaq Gold (Applied Biosystem) in a 50 *μ*l volume were performed with initial denaturation at 95°C for 10 min, followed by 35 cycles (95° for 30 s, 55°C for 30 s and 72°C for 45 s) and a final extension at 72°C for 10 min. The primer sequences were as follows for *hTERT* promoter (NG_009265): P1F 5′-TTTGCCCTAGTGGCAGAGAC-3′, P1R 5′-GCCGGAGGAAATTGCTTTAT-3′ P2F 5′-CTACTGCTGGGCTGGAAGTC-3′, P2R 5′-AGAAAGGGTGGGAAATGGAG-3′ and for *SMAD3* promoter (NG_011990): P1F 5′-CCAAGGTGGGAGGAATCAG-3′, P1R 5′-GAGTGCAATGGTGCCATCTT-3′ P2F 5′-CTTCTGGGCTGACTGTGGAT-3′, P2R 5′-CGACTAGCCGGTGTCTAAGC-3′. The primer sequences for *cMyc* promoter were described previously (Han *et al*, 2004). PCR products were fractionated on 1% agarose gel, and ethidium bromide-stained DNA was visualised on Ultraviolet Transilluminator (Spectroline, Westbury, NY, USA).

### Western blot analysis

Total protein were extracted from the tumour samples, normal renal cortical tissue and transfected TK-10 cells, using CHAPS lysis buffer (3-((3-cholamidopropyl) dimethyl-ammonio)-1-propane sulphonate). A total of 10 *μ*g of proteins were separated by 10% SDS polyacrylamide gel electrophoresis and transferred to nitrocellulose membrane (Hybond-ECL, Amersham Biosciences, Buckinghamshire, UK). Membranes were blocked in TBS containing 5% dried milk and 0.1% Tween-20 and probed with monoclonal mouse antibodies against WT1 (1 : 500; Dako, Glostrup, Denmark), cMyc (1 : 1000, Cell Signalling Technology Inc.) and *β*-actin antibodies (1 : 10 000, Chemicon International, Temecula, CA, USA). After a second incubation with peroxidase-conjugated anti-mouse or anti-rabbit antibodies (1 : 5000, Dako), proteins were visualised using an enhanced chemiluminescent detection system (ECL-advance, Amersham Biosciences).

### Immunofluorescence

At 48 h after transfection, cells were fixed with 3% formaldehyde and 2% sucrose in PBSA and then permeabilised with 0.1 M glycine. Cells were blocked with 2% normal goat serum and 0.4% Triton X-100 in PBSA for 30 min, followed by overnight incubation with mouse monoclonal WT1 antibody (1 : 100, Dako). After washing with 0.2% Triton X-100 in PBSA, cells were probed with secondary antibody Alexa Flour 488 rabbit anti-mouse IgG (H+L) in 1 : 500 concentration (Molecular Probes Inc., Eugene, OR, USA), and DAPI stained for nuclear visualisation. Images were captured using a NikonEFD3 microscope (Boyce Scientific, Gray Summit, MO, USA) and Nikon camera (100Eplan (160/0.17) objective; Nikon, Melville, NY, USA).

### Telomerase activity determination

Telomerase activity was evaluated using quantitative telomerase detection kit (QTD kit, Allied Biotech Inc, Ijamsville, CA, USA). A total reaction volume of 25 *μ*l consisted of 12.5 *μ*l of 2 × QTD pre-mix (provided by the kit), 250 ng protein CHAPS extract (supplemented with 1 U *μ*l^–1^ RNAsin and 1 mM DTT) and water. The qRT–PCR was performed on a 7000 sequence system (Applied Biosciences). Standard curve generated by TSR control template allows the calculation of the amount of template with telomeric repeat created by telomerase using 7000 SDS system software (Applied Biosystem).

### Statistical analysis

Statistical analysis was performed using SPSS (version 15, SPSS Inc., Chicago, IL, USA) statistical software. Mann–Whitney *U*-test was used to compare differences in the gene expressions of two independent variables. Correlations between two variables were tested according to Spearman's correlation test (statistical significance *P*⩽0.05).

## Results

### WT1 expression was inversely correlated to hTERT and cMyc in ccRCC

**Figure 1:**
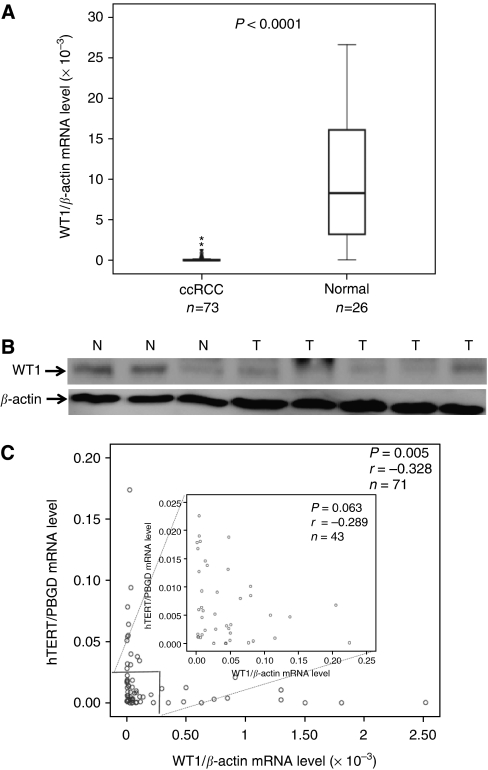
Wilms' tumour 1 (*WT1*) expression is negatively related to human telomerase reverse transcriptase (*hTERT*) mRNA levels in clear cell renal cell carcinoma (ccRCC). (**A**) Significant decrease in *WT1* mRNA expression in ccRCC compared with normal renal cortical tissue. (**B**) Western blotting shows decreased WT1 protein expression in four of five ccRCC tumour samples (T) compared with normal renal cortical tissue (N). (**C**) A reverse correlation exists between mRNA expression of *WT1* and *hTERT* in ccRCC. Samples with low expression levels for both *hTERT* (⩽0.025) and *WT1* (⩽0.25) also showed a similar trend.

*WT1* mRNA levels were analysed in 73 ccRCC specimens and 26 tumour-free renal cortical tissue samples using qRT–PCR. Significantly lower *WT1* RNA levels were found in the tumour samples in comparison with renal cortical tissue (*P*<0.0001), as shown in Figure 1A, indicating a downregulation of *WT1* in ccRCC. Immunoblotting for WT1 revealed lower protein levels in randomly selected tumour samples compared with tumour-free renal cortical tissues (Figure 1B)

We have previously demonstrated significantly higher mRNA levels of *hTERT* and *cMyc* in ccRCC compared with renal cortical tissue (Sitaram *et al*, 2009). In the present ccRCC samples, negative correlations were found between *WT1* and *hTERT* (*P*=0.005, *r*=−0.328, Figure 1C) and between *WT1* and *cMyc* (*P*=0.05, *r*=−0.246, *n*=64, not shown in figures). We could also demonstrate a tendency towards a negative association between *WT1* and *hTERT* for a subset of samples with lower expression levels for both *hTERT* and *WT1* (*P*=0.063, Figure 1C).

### WT1 expression was not associated with clinical features

*WT1* mRNA levels did not differ depending on patient age, gender, tumour grade or stage (*P*>0.05, for each parameter, data not shown). No significant difference in survival time was observed when the ccRCC cases were subdivided into two subgroups with a cutoff at the median *WT1* mRNA level (data not shown).

### Forced expression of WT1 can suppress hTERT and cMyc mRNA levels

**Figure 2:**
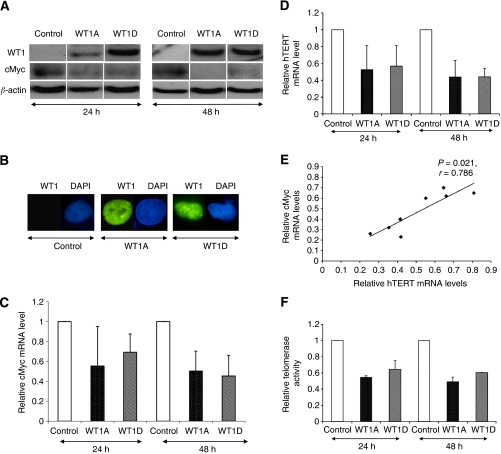
Forced overexpression of Wilms' tumour 1 (WT1) induces downregulation of human telomerase reverse transcriptase (*hTERT*) mRNA, telomerase activity and cMyc expression in TK-10 clear cell renal cell carcinoma (ccRCC) cell line. (**A**) Western blotting shows increased protein expressions of WT1 and reduced cMyc protein in WT1-transfected TK-10 cells. (**B**) TK-10 cells, which were transfected with WT1A or WT1D or pcDNA empty vector as control, demonstrate predominant nuclear localisation of WT1 protein (green) by immunostaining with anti-WT1. Nuclei were visualised by 4,6-diamidino-2-phenylindole (DAPI) (blue) staining. (**C**) Decreased *cMyc* mRNA levels in WT1-overexpressed cells but not in control cells. (**D**) Decreased *hTERT* mRNA levels in WT1-overexpressed cells but not in control cells. (**E**) A significant correlation between the degree of inhibition for *hTERT* and *cMyc* by forced overexpression of WT1A and WT1D is demonstrated by plotting the mean expression values in a series of separate transfections. (**F**) Decreased telomerase activity in WT1-overexpressed cells but not in control cells.

In order to answer whether the WT1 protein can function as a negative regulator of *hTERT* and/or *cMyc* transcription, we performed transfection experiments using TK-10 cells. High expression levels of WT1 isoforms A and D were demonstrated at 24 and 48 h after transfection (Figure 2A). It has been demonstrated that nuclear localisation of WT1 is a prerequisite for its transcriptional regulatory capacity (Ye *et al*, 1996). Therefore, immunostaining was performed 48 h after transfection verifying WT1 protein localisation in nuclei (Figure 2B). WT1A and WT1D overexpression induced a decrease in *hTERT* and *cMyc* mRNA levels as shown in Figures 2C and D. The downregulation of cMyc was also demonstrated by immunoblotting (Figure 2A). The repressive effects on *cMyc* and *hTERT* varied between experiments. By plotting the mean expression values for *hTERT* and *cMyc* in a series of separate WT1 transfections, we found a strong correlation, indicating a similar degree of inhibition (Figure 2E). This gives support for the idea that cMyc is involved in the *hTERT* regulation. Furthermore, both *WT1A* and *WT1D* transfections reduced telomerase activity after 24 and 48 h (Figure 2F).

### WT1 can regulate hTERT transcription via multiple pathways

**Figure 3:**
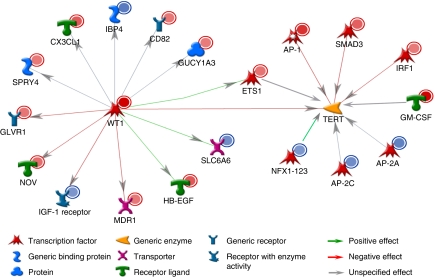
Altered gene expression of Wilms' tumour 1 (WT1) target genes and human telomerase reverse transcriptase (*hTERT*) transcriptional regulators by WT1 overexpression in TK-10 cells detected using expression array analysis. Network analysis was performed based on array data using the Metacore GeneGo software. Increased gene expression is indicated by a filled red circle on the upper right corner of each network object, whereas a blue dot indicates downregulation (⩾2-fold, *P*<0.01 compared with control). Gene/protein objects are represented by various shapes and colours depending on their functional annotations. Colours of the lines indicate inhibition (red) and activation (green), and gray arrows represent unspecified interactions (e.g., promoter binding).

**Figure 4:**
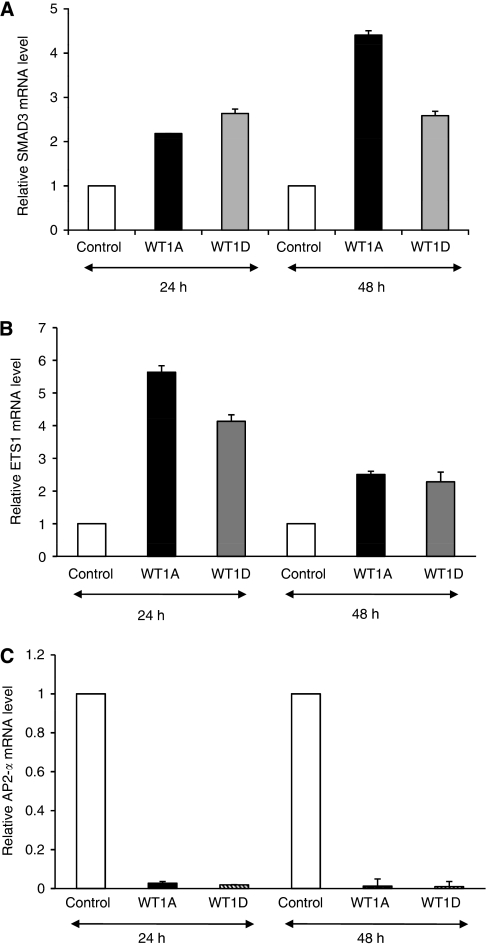
Validation of gene expression of *SMAD3* (**A**), *ETS1* (**B**) and *AP-2α* (**C**) by quantitative real-time PCR (qRT–PCR).

Gene expression alterations induced by WT1 transfection in TK-10 cells were detected using whole genome expression array analysis and several genes reported to be WT1 targets were found to be affected (Figure 3). Further analysis was focused on *hTERT*-regulating genes identifying an increased expression of several repressors. Fold changes, based on the array data, in expression of *hTERT* transcriptional regulators after WT1 transfection are summarised in Table 1. Two negative *hTERT* regulators, *SMAD3* and *JUN*, were significantly upregulated. Moreover, the *GM-CSF*, *ETS1* and *IRF1* genes also with suggested negative effects on *hTERT* transcription demonstrated increased expression. In contrast, *AP-2α*, *AP-2γ* and *NFX1*, all with potential positive effects on hTERT expression, were downregulated. The altered expression of *SMAD3*, *ETS1* and *AP-2α* was validated by qRT–PCR, showing a good correlation with the array data (Figure 4).

### WT1 binds to the hTERT, c-Myc and SMAD3 promoters

**Figure 5:**
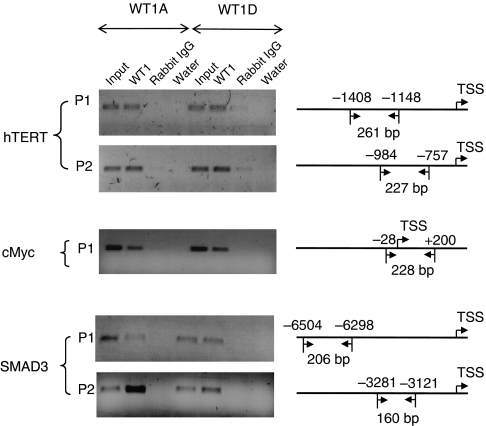
DNA binding of Wilms' tumour 1 (WT1) protein to the human telomerase reverse transcriptase (*hTERT*), *cMyc* and *SMAD3* promoters. Chromatin immunoprecipitation (ChIP)/PCR assay after forced overexpression of WT1A or WT1D in TK-10 cells as indicated. The location and size for each PCR product are illustrated on the right. TSS, transcriptional start site.

To demonstrate that WT1 proteins bind to the promoters of the *hTERT*, *cMyc* and *SMAD3* genes, we performed ChIP experiments. As shown in Figure 5, a single band was observed when chromatins from WT1-transfected TK-10 cells were immunoprecipitated with WT1 antibody. This was not observed when rabbit IgG antibody was used. By ChIP/PCR assay we could demonstrate direct binding of WT1 to the *hTERT*, *cMyc* and *SMAD3* promoters.

## Discussion

In this study, we describe a functional link between WT1 expression and the transcriptional activity of one of its target genes, *hTERT*, in human ccRCC. This link was identified through (1) an inverse relationship between *WT1* and *hTERT* gene expression levels in clinical ccRCC samples; (2) direct binding of WT1A and WT1D to the *hTERT* promoter; and (3) downregulation of the *hTERT* gene after experimentally induced WT1 overexpression. The collected data indicated that WT1 can control *hTERT* expression via multiple pathways and thereby act as a tumour suppressor in ccRCC.

WT1 is known to be a potent transcriptional regulator of many downstream targets, and can thus function as a tumour suppressor or an oncogene depending on cell type and tumour entity. Recent studies have demonstrated high expression of *WT1* in bone and soft tissue sarcomas, breast cancer and lung cancer (Loeb *et al*, 2001; Oji *et al*, 2002; Ueda *et al*, 2003). Therefore, the *WT1* gene has been proposed as an oncogene in these contexts. In contrast to these observations, we here found significantly lower *WT1* RNA levels in ccRCC samples compared with tumour-free renal cortical tissue. Only few studies have previously investigated *WT1* in human RCC. Using northern blot analysis, Campbell *et al* (1998) demonstrated aberrant *WT1* expression in four out of five RCC samples and in several RCC-derived cell lines arguing against *WT1* being a tumour suppressor in this tumour type. In contrast, later studies have generated data in accordance with our study. Niu *et al* (2005) showed low *WT1* transcription levels in ccRCC samples in comparison with tumour-free kidney tissue. By immunohistochemistry Nakatsuka *et al* (2006) found WT1 protein in four out of twelve (33%) ccRCC samples to be compared with >80% positivity in endometrial and brain tumours. On the basis of these reports and this study the collected data suggest that *WT1* can act as a tumour suppressor in ccRCC.

The transcriptional activity of the *hTERT* gene is the net result of many positively and negatively acting factors. WT1 has been identified as a cell type-specific *hTERT* transcriptional repressor, acting through WT1-binding site in the promoter (Oh *et al*, 1999). Our clinical data, showing a negative correlation between *WT1* and *hTERT* RNA levels, support the view that WT1 can suppress *hTERT*. In addition, the experimental cell line data further demonstrated that WT1 overexpression caused *hTERT* and telomerase repression. We could also show a direct binding of WT1 to the *hTERT* promoter in line with previous observations (Oh *et al*, 1999). Bellon and Nicot (2008) recently demonstrated that IL-2-induced upregulation of *hTERT* and telomerase activity in HTLV1-infected cells were due to sequestration of WT1 in the cytoplasm. In this study, we demonstrated nuclear localisation of WT1 protein after transfection, indicating that one prerequisite for its suppressive function was fulfilled.

One known activator of *hTERT* gene transcription is cMyc (Wu *et al*, 1999). In a previous study, we demonstrated a positive correlation between *hTERT* and *cMyc* in ccRCC (Sitaram *et al*, 2009). Cell line experiments showed that *hTERT* could be activated by a novel *PTEN* regulator, *DJ-1*, and *cMyc* seemed necessary for the upregulation (Sitaram *et al*, 2009). Studies of WT1 effects on the *cMyc* promoter have given divergent results indicating both stimulatory (Han *et al*, 2004) and repressive functions (Hewitt *et al*, 1995; Han *et al*, 2004). We observed a strong trend towards a negative correlation between *WT1* and *cMyc* RNA levels in the clinical ccRCC samples, and overexpression of WT1 did suppress *cMyc* at the transcriptional and protein level. Our observations indicate that loss of WT1 function in ccRCC can result in increased cMyc expression, which may contribute to *hTERT* expression. The repressive effects of WT1 on *hTERT* and *cMyc* expression seemed to be parallel in repeated experiments, further indicating that cMyc acts as a regulator of *hTERT* in ccRCC.

Microarray analyses have previously been used to identify *WT1* target genes (Kim *et al*, 2007). In this study, we found WT1 induced effects on a number of known targets for WT1, but also on *hTERT* transcriptional regulators previously not described as WT1 targets. Among negative *hTERT* regulators, *SMAD3* and *JUN* were strongly upregulated by *WT1* transfection. ChIP analysis revealed that WT1 can directly bind to the *SMAD3* promoter. Direct repression of *hTERT* by *SMAD3* via TGF-*β* signalling has been reported (Lacerte *et al*, 2008). JUN was originally thought to be identical to the transcription factor AP-1. However, it is now known that AP-1 constitutes a group of dimeric basic region-leucine zipper proteins that belong to the FOS, MAF and ATF subfamilies. JUN is the most potent transcriptional activator in this group of proteins (Hattori *et al*, 1988). Previous studies have shown that overexpression of AP-1 can downregulate *hTERT* transcription in cancer cells (Takakura *et al*, 2005). The combination of c-Fos/c-Jun or c-Fos/JunD strongly suppresses *hTERT* promoter activity in transient-expression experiments (Takakura *et al*, 2005).

*IRF1* has been shown to be important in apoptosis and cell differentiation (Harada *et al*, 1993). A previous study demonstrated that IRF1 is a mediator for interferon-*γ*-induced inhibition of *hTERT* expression and telomerase activity in cervical cancer cells (Lee *et al*, 2003). In this study we observed about two-fold increased expression of *IRF1* by WT1D, indicating that it might have a negative effect on *hTERT* promoter activity. The clinical and experimental data presented here strongly suggest that WT1 contributes to *hTERT* inactivation, directly by acting on the promoter and indirectly via *hTERT*-negative regulators such as SMAD3 and JUN.

GM-CSF can regulate *hTERT* transcription both positively and negatively, but only in combination with other genes (Mano *et al*, 2000). *GM-CSF* was strongly upregulated by WT1 in our analysis, indicating that it might act as an *hTERT* repressor. Further, we also found that WT1 overexpression induced increased *ETS1* expression. Transcriptional activation of *ETS1* by WT1 has been reported in tumour vascularisation via regulation of endothelial cell proliferation and migration (Wagner *et al*, 2008). The role of ETS proteins, especially ETS1 and ETS2, in telomerase regulation seems to depend on ETS-binding sites in the *hTERT* promoter and protein–protein interactions (Dwyer *et al*, 2007). Xiao *et al* (2003) demonstrated an inhibitory as well as an activating effect on *hTERT* transcription mediated through different ETS-binding sites. In this study, increased *ETS1* and reduced *hTERT* expression by WT1 may suggest a negative role of ETS1 in *hTERT* transcriptional regulation in ccRCC.

Two isoforms of NFX1 have been identified, the longer 1120-amino acid isoform as NFX1-123 and the shorter 833-amino acid isoform as NFX1-91 (Gewin *et al*, 2004). NFX1-123 was demonstrated to co-activate the *hTERT* promoter with cMyc, whereas NFX1-91 repressed the *hTERT* promoter (Gewin *et al*, 2004). Significant reduction of *NFX1-123* expression by WT1 was found in our array analysis, suggesting that it might act as an *hTERT* activator in ccRCC. The protein AP-2*β* has been identified as a transcriptional activator of the *hTERT* promoter in human lung cancer cells (Deng *et al*, 2007). The family of AP-2 proteins consists of five different transcriptional factors (*α*, *β*, *γ*, *δ* and *ε*), encoded by separate genes and with different biological functions. The AP-2-binding site at nucleotides –129 to –121 in the *hTERT* promoter is essential for transcriptional regulation during differentiation of human sarcoma cells (Ma *et al*, 2003), but the role of individual AP-2 family members has not been detailed. In this study *AP-2α* and *AP-2γ* were downregulated by WT1, suggesting that WT1 thereby can affect *hTERT* regulation.

Interestingly, in T-cell cultures we observed that upregulation of telomerase was associated with decreased expression of *GM-CSF*, *JUND*, *ETS1*, *SMAD3* and increased expression of *AP-2α* and *cMyc* (Degerman *et al*, 2010). The collected data strengthen the scenario of *hTERT* regulation via multiple pathways.

By gene expression array analysis, we found that forced expression of WT1 had a regulatory function on many known WT1 target genes. However, the effects on transcription of some target genes were different from previously published data in other cellular systems, further supporting the view of cell type specificity regarding WT1 function. In conclusion, this study strongly suggests that *WT1* can act as a tumour suppressor in ccRCC-regulating *hTERT* gene expression via multiple pathways.

## Figures and Tables

**Table 1:** Fold induction in expression of hTERT transcriptional regulator genes in response to forced WT1 expression in TK-10 cells by microarray analysis

				**Fold change (*vs* control)**			
				**WT1A**		**WT1D**	
**Gene name**	**Synonyms**	**Entrez ID**	**Expected effect on hTERT**	**24 h**	**48 h**	**24 h**	**48 h**
*CSF2*	GM-CSF	1437	Activator/repressor	19.2^*^	18.7^*^	15.8^*^	12.9^*^
*ETS1*	ETS1	2113	Activator/repressor	3.5^*^	2.7^*^	3.7^*^	2.5^*^
*IRF1*	IRF1	3659	Repressor	1.4	1.9	1.7^*^	2.6^*^
*JUN*	AP1	3725	Repressor	12.8^*^	6.4^*^	11.9^*^	5.7^*^
*SMAD3*	SMAD3	4088	Repressor	6.3^*^	3.1^*^	4.6^*^	3.5^*^
*NFX1*	NFX-123	4799	Activator	−2.5^*^	−2.0^*^	−2.1	−2.0^*^
*TFAP2A*	AP-2*α*	7020	Unspecified	−6.8^*^	−8.1^*^	−6.8^*^	−8.0^*^
*TFAP2C*	AP-2*γ*	7022	Unspecified	−3.5^*^	−5.0^*^	−3.6^*^	−5.4^*^

Abbreviations: hTERT=human telomerase reverse transcriptase; WT1=Wilms' tumour 1.

^*^Statistical significance (*P*<0.01).
